# Supplementary material for: Mitochondrial sAC-cAMP-PKA Axis Modulates the ΔΨm-Dependent Control Coefficients of the Respiratory Chain Complexes: Evidence of Respirasome Plasticity
Source: Int J Mol Sci. 2023 Oct 13;24(20):15144. doi: 10.3390/ijms242015144 (PMC10607245; doi:10.3390/ijms242015144)
Supplement: Supplementary file 1 [file ijms-24-15144-s001.zip › ijms-2661306-SI.pdf]

Supplemental Fig. S1

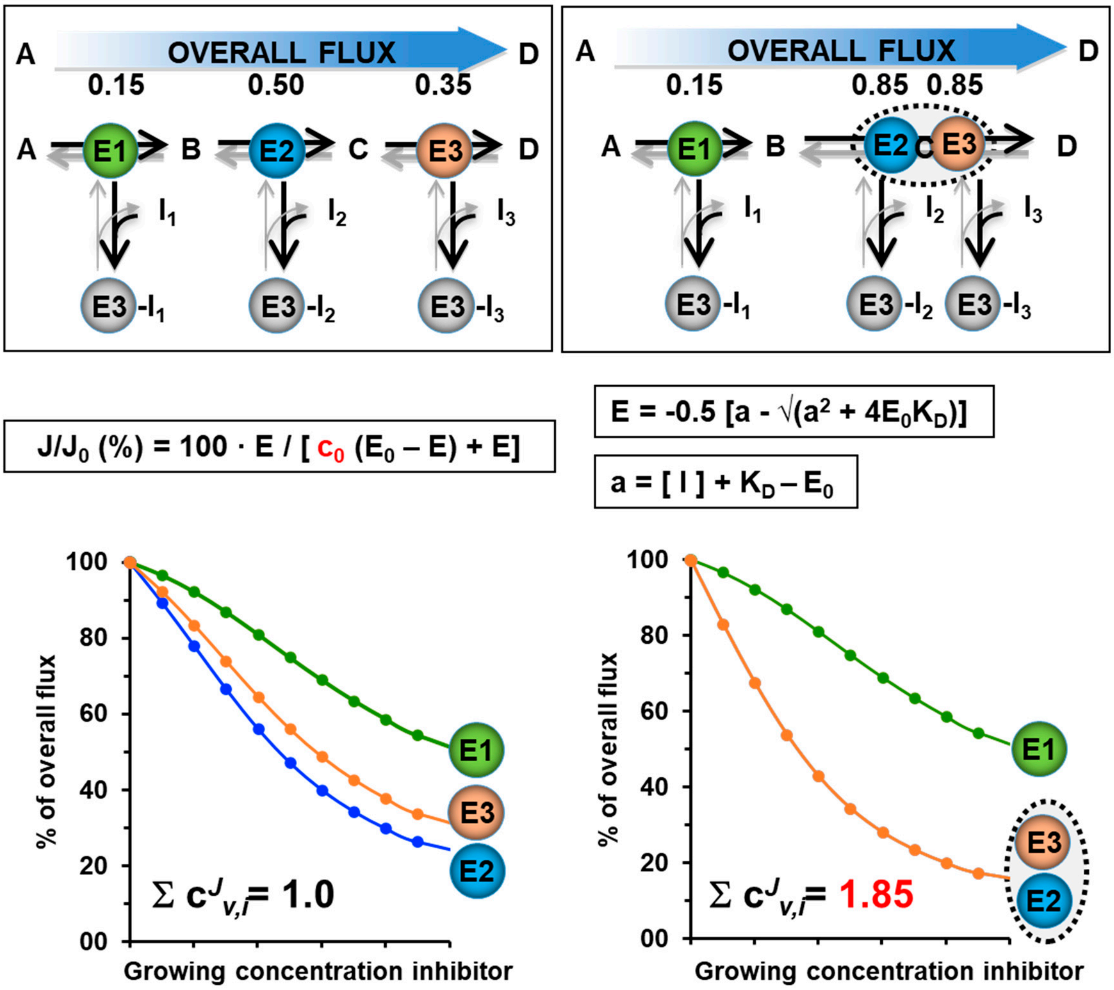

Figure S1. Schematic representation of the distribution of the control coefficients in a multi-enzymatic metabolic pathway. An overall flux from an initial compound A to the final compound D is shown as resulting from the linear sequence of the three enzymatic steps E1, E2, E3 each specifically inhibitable by I_1, I_2 and I_3 respectively. The assumed control coefficient for each of the individual steps is given as figures on the top of them. The graphs show a simulation of the inhibitory titration curve for each of the three enzymatic steps as percentage of the uninhibited flux at increasing concentrations of the specific inhibitor. The equation used is indicated and it is from [Gellerich FN 1990 ref. 25 in the main text] as modified in [Quarato G 2011 ref. 18 in the main text]. Similar arbitrary values for E_0 (total concentration of the enzyme) and for K_D (dissociation constant of the E-I complex) were used for the three enzymes whereas different values of c_0, as indicated, were imputed in the equation. Two conditions are set: the left part assumes that the three enzymes are functionally independent; the right part assumes that two of the three steps are complexed (E2-E3). The sum of the control coefficients is shown in the graphs for the two conditions. See the main text and further references therein regarding the "metabolic flux theory".
